# Evaluation of Process Indicators and Challenges of the Elimination of Mother-to-Child Transmission of HIV, Syphilis, and Hepatitis B in Bali Province, Indonesia (2019–2022): A Mixed Methods Study

**DOI:** 10.3390/tropicalmed8110492

**Published:** 2023-11-03

**Authors:** Luh Nik Armini, Elsa Pudji Setiawati, Nita Arisanti, Dany Hilmanto

**Affiliations:** 1 Doctoral Study Program, Faculty of Medicine, Universitas Padjadjaran, Sumedang 45363, Indonesia; luh14001@mail.unpad.ac.id; 2 Midwifery Science Program, Faculty of Medicine, Universitas Pendidikan Ganesha, Bali 81116, Indonesia; 3 Department of Public Health, Faculty of Medicine, Universitas Padjadjaran, Sumedang 45363, Indonesia; nita.arisanti@unpad.ac.id; 4 Department of Child Health, Faculty of Medicine, Universitas Padjadjaran, Sumedang 45363, Indonesia; danyhilmanto@yahoo.com

**Keywords:** EMTCT, hepatitis B, HIV, pregnant women, syphilis

## Abstract

Background: This study was conducted to describe the prevalence of and evaluate the processes and challenges in implementing the elimination of HIV, syphilis, and hepatitis B transmission from mother to child in Bali Province, Indonesia. Methods: The research method used is a descriptive approach using indicators and a set of processes by the WHO, quantitative methods using descriptive analysis, and qualitative methods using phenomenological paradigms through in-depth interviews and FGD with healthcare professionals involved in the elimination of mother-to-child transmission (EMTCT) program. Results: The indicators that have successfully met the target for 4 years are antiretroviral therapy (ART) coverage in Badung District (≥95%) and ANC coverage (at least one visit) in Buleleng District (≥95%). The study found low prevalence rates of HIV, syphilis, and hepatitis B among pregnant women in the three districts. There are some indicators that show improvement from 2019 to 2022, namely, syphilis (60.44% to 86.98%) and hepatitis B (29.03% to 95.35%) screening coverage showed improvements, with increasing screening rates observed in Buleleng District. However, adequate treatment coverage for pregnant women with syphilis decreased in Denpasar City in 2022 compared to 2019 (100% to 71.28%). Despite data on hepatitis B treatment being unavailable, hepatitis B vaccination coverage exceeded the WHO target in all three districts. The utilization of the information system is not yet optimal, and there is a lack of ability to track cases. Furthermore, there is insufficient involvement of the private sector, particularly in screening, and a lack of standardized procedures in the management of referrals for pregnant women with hepatitis B. Conclusion: The prevalence of HIV, syphilis, and hepatitis B among pregnant women has consistently remained below the Ministry of Health’s target for four years. Despite this, there are a lot of targets, and the indicator EMTCT process has yet to reach the WHO target. The challenges for each district in reaching the WHO target include providing syphilis and hepatitis B reagents and benzatine penicillin; increasing private sector involvement; and strengthening information systems, policies, and guidelines for the management of hepatitis B among pregnant women in line with WHO recommendations to achieve EMTCT.

## 1. Introduction

Through the World Health Organization (WHO), the global community is committed to eliminating the transmission of HIV, syphilis, and hepatitis B from mother to child, as they pose a public health threat worldwide. This effort is known as the triple elimination of mother-to-child transmission (EMTCT) of human immunodeficiency virus (HIV), syphilis, and hepatitis B [1]. These three infectious diseases rank the highest among infections transmitted from infected mothers to infants during pregnancy, childbirth, and breastfeeding, particularly in low- and middle-income countries, such as those in Asia and Africa [2,3]. Globally, it is estimated that every year, 1.3 million pregnant women are living with HIV, and if not treated, they can transmit the infection to approximately 15–45% of their babies [4]. The highest mortality due to HIV infection in children occurs within the first few months of life [5]. Syphilis is a sexually transmitted infection (STI) that, when left untreated, becomes the leading cause of syphilis transmission from a mother to her unborn child (congenital syphilis/CS). It is the second leading cause of stillbirth, following malaria, which is associated with preterm birth, low birth weight, miscarriage, and even infant death [6,7,8]. Infants born with CS are twice as likely to expire compared to those born without CS [9]. Hepatitis B is caused by the hepatitis B virus (HBV), which leads to liver infection that can become chronic and is associated with a high risk of death from cirrhosis and liver cancer. Infants born to mothers infected with hepatitis B that do not receive immediate vaccination after birth are at risk of contracting hepatitis B. Approximately 95% of infected infants are at risk of developing chronic hepatitis and becoming a transmission source to other infants [10].

In 2020, there was a four-fold increase in new cases of AIDS infection in children in the world compared to the established target [1]. There is also a gap between screening and treatment for pregnant women with hepatitis B and a slow decline in CS [1,11,12]. The WHO’s regional office for the Southeast Asia region developed “The Regional Framework for the Triple EMTCT in Asia and the Pacific 2018–2030”, aiming to achieve zero infection in children by 2030 [2]. In line with this, the WHO has also released the third edition of global guidelines on the criteria and process for validating the elimination of HIV, syphilis, and hepatitis B transmission from mother to child, providing standardized targets and criteria for assessing the progress of countries in achieving single or triple elimination efforts [13]. Triple elimination also aims to reduce these diseases’ incidence, prevalence, morbidity, and mortality [13].

Several countries in the Southeast Asia region, such as Thailand, Malaysia, The Maldives, and Sri Lanka, have successfully achieved the elimination of HIV and syphilis transmission from mother to child [14]. Like other countries, Indonesia is also making efforts to eliminate the transmission of these three infections from mother to child. The implementation of triple elimination in Indonesia is regulated by the Ministry of Health of the Republic of Indonesia.

The triple EMTCT in Indonesia is expected to be achieved by the designated year of 2022, known as the elimination year. The Ministry of Health of the Republic of Indonesia expects that by the elimination year, at least one district or one province will be able to declare the EMTCT [15]. In Indonesia, the policy for testing EMTCT is governed by relevant legislation. This policy ensures that screening procedures are integrated into antenatal care (ANC) services, known as integrated ANC, enabling the early detection and prevention of HIV, syphilis, and hepatitis B transmission from pregnant mothers to their infants. Additionally, the procurement of necessary reagents for these tests is regulated within the national budget, ensuring the availability of facilities and resources required to effectively implement the EMTCT program [15].

Several studies have been conducted in Indonesia, specifically in Kupang and Surabaya, focusing on the achievement of screening, treatment, and HIV prevalence among pregnant women [16,17]. Although policies and strategies for achieving triple elimination are in place and implemented by the Ministry of Health, there still needs to be studies reporting on the achievement of triple EMTCT at the national, provincial, and district levels. This study aims to evaluate the process indicators and challenges in implementing the EMTCT at the district level in Bali Province. Bali Province has its own distinct cultural, social, and healthcare context. Studying the elimination efforts in this region allows for a deeper understanding of the challenges and barriers that may be specific to the local context. This knowledge can help tailor interventions and strategies to address the population’s unique needs. By addressing these aspects, the study provides valuable information for policymakers, healthcare providers, and stakeholders involved in efforts to achieve EMTCT. It offers insights into the progress made, challenges faced, and areas that require attention, ultimately contributing to developing targeted interventions and strategies for achieving successful elimination outcomes.

## 2. Methods

### 2.1. Study Design

The research was conducted using an explanatory mixed method. This approach involved conducting a quantitative study as the initial phase, followed by a qualitative study. The explanatory study aimed to strengthen the findings of the quantitative study conducted in the first phase by conducting a qualitative study in the subsequent phase. The data for this research were collected from August 2022 to February 2023.

### 2.2. Study Setting

Bali Province ranks sixth regarding the highest number of HIV cases in Indonesia. For the research, three districts were selected as the study locations: Denpasar City, Badung District, and Buleleng District. These districts were chosen as the pilot project sites for the PMTC program in Bali Province, and they have the highest number of pregnant women as the target population each year.

### 2.3. Participants and Recruitment

The qualitative participants included 36 health workers from primary healthcare centers, the District Health Office, and government-owned hospitals. Given the significant role of the public sector in the triple elimination program, this sector was specifically chosen. Focus group discussions (FGDs) were conducted four times, with each FGD involving separate groups of participants. The FGD participants comprised a total of 30 individuals, distributed across six doctors, ten midwives responsible for maternal health programs at primary healthcare centers (PHC), seven nurses, and seven laboratory technicians. To ensure comprehensive representation and dynamic discussions among the healthcare professionals involved in the efforts to eliminate the transmission of HIV, syphilis, and hepatitis B from mother to child, each FGD consisted of 6–8 individuals. The determination of the number of participants for both FGDs and interviews resulted from collaborative discussions between the researchers and the District Health Office. In addition to the FGDs, in-depth interviews were conducted with six individuals. Three were responsible for maternal and child health programs at each health office, while the other three were in charge of programs at advanced-level referral hospitals dedicated to treating pregnant women with HIV, syphilis, and hepatitis B within each district.

### 2.4. Process Indicator and Measurement

The selection of process indicators and their measurement followed the Global Guidance on Criteria and Processes for Validation of EMTCT of HIV, Syphilis, and Hepatitis B Virus, as outlined by the WHO third edition (see Table 1) [13].

### 2.5. Data Collection

The quantitative study was conducted retrospectively using secondary data from 2019–2022. The data were descriptively presented to illustrate the coverage of each EMTCT process target, maternal treatment, and infant HBV vaccination according to the minimum validation criteria set by the WHO. The data were obtained from reports on eliminating HIV, syphilis, and hepatitis B transmission from mother to child from each district health office, mother–baby cohorts, antenatal care service registers at each PHC, and immunization reports for infants/children at the district level. The data obtained were a combination of services provided by public and private healthcare facilities, reported by the responsible midwives in charge of maternal and child health programs in their respective areas to the district health offices every month. The number of pregnant women who were registered for antenatal care visits from the secondary data in the 2019–2022 cohort is as follows: in Denpasar, ranged from 17,306 to 18,161 per year; in Badung, from 10,084 to 12,358 per year; and in Buleleng, ranged from 10,676 to 12,259 per year.

### 2.6. Data Analysis

The quantitative analysis used a descriptive analysis and was performed to evaluate the process indicators from 2019 to 2022. The qualitative data analysis was conducted using a thematic content analysis approach with the assistance of Nvivo 12 software (QSR International Pty Ltd., Version 12, 2018). The raw data in the transcriptions were processed and analyzed by assigning codes until thematic patterns emerged. The coding process was conducted deductively, adopting themes validated by the WHO.

### 2.7. Ethical Clearance

The Health Research Ethics Committee of Universitas Padjadjaran approved the study in Bandung, Indonesia (Approval No. 798/UN6.KEP/EC/2022). All the qualitative participants received information about the research, including the research questions, objectives, and data publication. Written consent was obtained from all the participants involved in the FGDs and in-depth interviews. The quotation text and article writing do not include the names of the participants to ensure confidentiality and anonymity.

## 3. Results

### 3.1. Distribution of Triple Infection

The prevalence of HIV, syphilis, and hepatitis B among pregnant women was low in three districts and decreased from 2019 to 2022. The average prevalence of pregnant women infected with HIV in the past four years was highest in Buleleng Regency at 0.33%, slightly higher than the target set by the Ministry of Health (0.30% each year). The average prevalence of pregnant women with syphilis was highest in Badung Regency at 0.70%, still below the target set by the Ministry of Health, which is 1.70%. The average prevalence of pregnant women with hepatitis B was highest in Buleleng Regency at 1.54%, which is still below the maximum target set by the Ministry of Health at 7.10% (see Table 2).

### 3.2. EMTCT Process (Coverage)

The achievement levels for reaching targets related to eliminating new infections and maternal treatment for HIV, syphilis, and hepatitis B in the three districts in Bali Province are still below the WHO standards, although some progress has been observed in certain aspects. Denpasar District had a change over time in HIV reaching 94.84% in 2022 compared to 2019, and there was a decrease in meeting the targets for almost all the EMCTC process indicators and ART as maternal treatment. Badung District has achieved the 100% target in ART coverage. Nevertheless, challenges persist in reaching the targets within all the EMCTC process indicators. Buleleng District has achieved successes, including reaching targets for ANC coverage and infant HBV vaccination. The coverage of syphilis and HBsAg antenatal testing increased in 2022 compared to 2019 (reaching 86.98 and 95.35, respectively), but it is still below the WHO target, especially the coverage of screening hepatitis B (<90%). However, there is a pressing need to improve all the indicators, which remain below their targets. The target of hepatitis B vaccination to prevent MTCT in the three districts from 2019 to 2022 has surpassed the WHO target (≥90%) for four consecutive years. It needs to be highlighted that data on hepatitis B treatment with high viral loads are unavailable and that hepatitis B vaccination coverage exceeded the WHO target in all three districts. (See Table 3).

### 3.3. Challenges of Triple EMTCT

The qualitative findings are presented in four aspects, aligning with the validation assessment by the WHO, which are further divided into eight themes: monitoring and evaluation, information systems, laboratory availability, data output, availability of reagents and drugs, policies, involvement of the private sector, and human rights. The themes and corresponding quotations can be found in Table 4.

#### 3.3.1. Monitoring and Evaluation

The district and provincial health offices conduct monitoring and evaluation of service implementation and report data by primary healthcare facilities and referral facilities every three months. Monitoring and evaluation meetings are conducted between program managers at the district level and maternal and child health program managers at the PHC and hospitals.

#### 3.3.2. Information Systems

The recording and reporting of data are accomplished using various information systems, including the HIV/AIDS and Syphilis Information System (SIHA), Hepatitis Information System (SIHEPI), Immunization Information and Data System (SIDI), and maternal health services reported through an e-cohort, which services providers in both the public and private sectors. Although information systems are available, not all of them function properly. The utilization of an e-cohort and SIHEPI has yet to be optimized since they were newly introduced in early 2022. The systems often encounter errors, and not all healthcare providers input the data, resulting in the need for manual recording.

#### 3.3.3. Data Sources

The reported data includes services provided by public and private healthcare facilities, such as independent midwife practices, specialist doctor practices, general practitioners, and private hospitals.

#### 3.3.4. Resources

Each primary healthcare center in the three districts has a medical laboratory technician responsible for conducting RDT (rapid diagnostic test) examinations. The healthcare centers have healthcare personnel consisting of doctors, midwives, nurses, and medical laboratory technicians. All the counselors have received training as counselors.

#### 3.3.5. Laboratory Availability

Each primary healthcare center serving as a screening site for HIV, syphilis, and hepatitis B in pregnant women has a laboratory capable of screening using RDT. Virology examinations can only be performed at advanced referral hospitals.

#### 3.3.6. Availability of Reagents and Drugs

The availability of reagents and drugs for HIV testing and treatment is consistently sufficient each year. The same applies to vaccines for HBIg (hepatitis B immunoglobulin) and hepatitis B immunization up to the third dose. However, there is often a shortage of reagents for syphilis and hepatitis B testing and benzathine penicillin. The insufficient availability of these items leads to variations in the coverage of the three infectious disease screenings and challenges to achieve EMTCT processes.

#### 3.3.7. Policies

The EMTCT policy in Indonesia is regulated by Ministerial Regulation No. 57 of 2017 and the Prevention of HIV, Syphilis, and Hepatitis B Transmission from Mother to Child Program Guidelines of 2019, published by the Ministry of Health, which align with WHO recommendations. All the healthcare facilities adhere to the national policy. Pregnant women with HIV are required to receive ART, syphilis treatment through benzathine penicillin injections, and hepatitis B treatment through referrals to specialists in internal medicine; however, not all PHCs refer cases to internal medicine or hospitals. Referrals to internal medicine or hospitals are only made for pregnant women infected with HBV who exhibit clinical symptoms of hepatitis.

#### 3.3.8. Involvement of the Private Sector

The involvement of the private sector, especially in maternal screening, is minimal. All the maternal screenings are conducted at public healthcare centers, so pregnant women seeking prenatal care in the private sector need to be referred to healthcare centers, resulting in longer testing times. Pregnant women with HIV and syphilis are required to be referred to government-owned hospitals for delivery. In contrast, those with hepatitis B can be referred to private and government hospitals.

#### 3.3.9. Human Rights

Verbal consent is obtained from pregnant women undergoing HIV, syphilis, and hepatitis B testing. Still, detailed information about the specific examinations is not provided initially to avoid potential patient rejection. Detailed information is provided if they are confirmed or tested positive. When a positive result is obtained, the women are directed to counseling rooms or VCT (voluntary counseling and testing) centers for counseling by trained healthcare professionals. There are forms for refusal and consent for testing and treatment for infected pregnant women.

## 4. Discussion

### 4.1. Principal Findings

The EMTCT program in Indonesia started in 2018 and has been ongoing for nearly five years. Countries typically take several years to eliminate HIV, syphilis, and hepatitis B transmission from mother to child [18,19,20]. This study found several key findings: (1) the prevalence of pregnant women infected with HIV, syphilis, and hepatitis is different across the districts but generally remained below the target the Ministry of Health set. (2) The coverage of syphilis and hepatitis B screening in pregnant women showed an increasing coverage in only one district but decreased in another. (3) The coverage of at least one antenatal care visit met the WHO target in two districts but fell below the target in one district. (4) The coverage of HIV testing in pregnant women exceeded the WHO target in one district. (5) ART treatment coverage for women with HIV met the criteria WHO for four years in only one district. Adequate treatment coverage for pregnant women with syphilis decreased in two districts. (6) Hepatitis B vaccination coverage met the WHO target in all three districts. (7) The challenges included mobility issues, COVID-19’s impact, reagent and drug availability, policy adherence, and limited involvement of the private sector.

In comparing our study with the previous study conducted by Wardiana et al. [21] in the east part of Indonesia, both investigations share a common focus on EMTCT in Indonesia; however, our study offers a more extensive perspective, as it encompasses data spanning nearly four years, providing a longer-term assessment of the EMTCT program’s progress. While both studies identify the disparities in the prevalence, screening coverage, and treatment, our research goes a step further by shedding light on the specific challenges faced during the implementation of the program, such as mobility issues, the impact of COVID-19, and policy adherence. This deeper exploration of the challenges adds valuable context and can inform more targeted interventions to enhance the program’s effectiveness. Moreover, a previous study was conducted in a tertiary hospital in 2018, when the program was still in its early stages or in the initiation phase. Additionally, nowadays, screening and treatment have already been conducted by PHC.

In comparison to several countries in the Southeast Asia region, including Thailand, Malaysia, The Maldives, and Sri Lanka, where the successful elimination of HIV and syphilis transmission from mother to child has been achieved, our study’s results indicate that Indonesia has not yet reached its elimination targets. These countries have demonstrated significant progress in their efforts to prevent the transmission of these infections to newborns. The key indicator for elimination, as defined by the World Health Organization (WHO), is the sustained achievement of the targets for three consecutive years.

The achievement of the process indicators, such as the minimum coverage of at least one ANC visit during pregnancy, screening for syphilis and hepatitis B has decreased in Denpasar and does not yet meet the minimal WHO criteria. The lack of availability of syphilis and hepatitis B reagents at primary healthcare facilities and the lack of private sector involvement are challenges to achieving targets according to WHO criteria. Several studies also found that high mobility among pregnant women in urban areas poses a barrier to accessing maternal and child health services, mainly PMTCT services [22]. The COVID-19 pandemic has also affected pregnant women who are migrant populations in accessing ANC services [23]. However, there has been an improvement in the screening coverage for syphilis and hepatitis B in the Buleleng district. According to several studies, factors such as the quality of prenatal care, maternal knowledge, and the availability of resources including healthcare providers, laboratories, medications, free financing, and reasonable access to healthcare services support the achievement of screening coverage for syphilis and hepatitis B in pregnant women [24,25,26,27]. High screening coverage and adequate treatment can reduce the transmission of infections from mother to child [28]. The integration of the elimination of these three diseases into maternal and child health services has proven to be cost saving and economically effective [29].

The treatment indicator for pregnant women with a stable HIV infection that has consistently met the minimum WHO target for four consecutive years is the Badung District. Learning from successful elimination efforts in countries like Cuba and Thailand, integrating PMTCT services into maternal and child health services, providing free pregnancy examinations and testing for partners, and offering free formula milk to infants exposed to HIV from their mothers have been implemented. Thailand has even engaged the private sector in the education and promotion of HIV/AIDS prevention [20]. Various successful efforts to initiate and maintain HIV treatment for mothers include partner support, family support groups, mentor mother programs, and eliminating stigma and fear. These factors can be optimized and applied in the two districts that have not yet achieved their targets, thus enabling the coverage of HIV treatment in pregnant women to reach the minimum target recommended by the WHO or expected by the Ministry of Health [19,30,31,32]. Maintaining HIV treatment for pregnant women requires additional efforts, such as assistance from NGOs and home visits by village midwives or designated midwives. Lack of information and stigma from family, society, and healthcare providers, as well as a lack of openness towards partners and family remain barriers for women living with HIV to initiate and sustain treatment [33,34,35].

Treatment coverage for pregnant women with syphilis has declined in Denpasar and Badung District. A limited supply of benzathine penicillin is a challenge to achieve adequate treatment among pregnant women with seropositive syphilis, thus pregnant women with seropositive syphilis must be referred to the hospital. Several studies have found that stigma related to treatment during pregnancy, poverty, citizenship status, lack of health literacy regarding sexually transmitted infection transmission, late screening (near delivery), and delayed treatment are some hindering factors in the screening and treating of pregnant women with syphilis [36,37].

The coverage of hepatitis B immunization has been established and meets the WHO indicators in all three districts for the last four years. However, there is a lack of data or reports regarding treatment for pregnant women with high viral loads (>200,000). This is because not all primary health centers recommend treatment to internists for pregnant women with hepatitis B, and management of hepatitis B among pregnant women was not in line with WHO guidelines. This aligns with research conducted in California, which found that only 60.9% of obstetricians recommended treatment to internists for pregnant women with HBV [38]. Treatment guidelines for pregnant women with hepatitis B in the three districts include referrals to internists, and there is currently no request for reports on treatment for pregnant women with hepatitis B from the Ministry of Health, leading to a lack of data on the treatment of infected pregnant women. Therefore, it is necessary to align perceptions or update local and national recommendations regarding managing infected pregnant women to adhere to WHO guidelines [39]. Tenofovir has been proven to prevent mother-to-child transmission of hepatitis B, reduce viral load, and have no adverse effects on either the mother or the baby [40]. The high cost of viral load testing for HBV, which is not covered by health insurance, remains a barrier to conducting viral load testing for pregnant women with HBV in Indonesia, especially in the three districts in Bali. This is consistent with research conducted in the United States, which found a gap between maximum testing for pregnant women and minimal management (approximately 7%) for pregnant women with HBV [12]. A study conducted in China found that EMTCT for HBV could be achieved even without HBV DNA testing; however, further surveys regarding hepatitis B prevalence in children under five are needed [41]. The coverage of hepatitis B immunization for infants already exceeds the target set by the WHO. Similarly, the administration of HBIg to infants born to mothers infected with hepatitis B, aiming to prevent mother-to-child transmission, is in line with WHO recommendations [39].

Overall, the screening coverage is lower in Denpasar for all three infections. Urbanization, high population mobility, and the presence of many newcomers can make patient follow-up and tracking more challenging. In some cases, newcomers who may not have easy access to healthcare services or who frequently change residences could be a vulnerable group in terms of reduced screening coverage. The high population mobility in Denpasar could be one of the reasons for the decline in screening coverage. Furthermore, facilitating easily accessible screening services and integrating healthcare systems to monitor patients with high mobility are needed. Identifying and addressing factors will be a crucial step in improving screening coverage and the success of the elimination program in Denpasar.

The prevalence of pregnant women with HIV, syphilis, and hepatitis B has decreased; however, the target is still below the ministry of health target. This aligns with the expected results of the Southeast Asia region of WHO, which aims to reduce the incidence and prevalence of pregnant women with HIV, syphilis, and hepatitis B [2]. WHO also evaluated the impact target for HIV, syphilis, and hepatitis B; however, the impact cannot be evaluated due to the lack of data of follow-ups of an exposed baby. Therefore, strengthening the health system to provide sustainable EMTCT services and meet the established validation standards set by WHO is essential. Private sector involvement is still minimal compared to the public sector. For example, a study conducted in Denpasar and Badung in 2010 found that only 50% of pregnant women referred to primary health centers for HIV screening were tested [42]. This finding is consistent with research conducted in North Sulawesi, which found minimal private sector involvement compared to the public sector [43]. However, the situation may differ in the three districts. Some primary health facilities charge fees for screening patients without health insurance and only the reagents are provided free of charge due to budget support from the government. In contrast, some primary health centers waive the fees altogether. This shows that policies vary among primary health centers regarding service flow for screening, highlighting the need for written standard operating procedures or local regulations governing the financing of HIV, syphilis, and hepatitis B screening for pregnant women.

### 4.2. Implications for Practice

The findings from the study have significant implications for improving practice and achieving the EMTCT of HIV, syphilis, and hepatitis B in Bali, Indonesia. Firstly, it is crucial to ensure that appropriate interventions are tailored to the specific needs of each area. Efforts should focus on increasing the coverage of syphilis and hepatitis B screening in pregnant women, particularly in districts where the rates have decreased. Additionally, enhancing the coverage of at least one antenatal care visit, HIV testing, and ART treatment for women with HIV is essential. Health systems need to be strengthened to achieve sustainable EMTCT services, and private sector involvement should be expanded. It is imperative to overcome challenges such as mobility issues, COVID-19’s impact, resource availability, data recording, laboratory capacity, reagent and drug availability, policy adherence, stigma, and access to information. By addressing these issues and implementing evidence-based strategies, Bali can work towards meeting WHO standards and ensuring mothers’ and their children’s health and wellbeing.

In practical terms, in addressing the challenges related to EMTC, it is imperative to explore avenues for securing the necessary resources to ensure effective management of hepatitis and syphilis cases among pregnant women. Currently, the funding relies on the national budget, hence the need for alternative funding sources. Considering the lack of available funds, it is becoming increasingly important to seek support from other governmental entities. Specifically, collaboration with the provincial government (regional budget allocation) could provide a viable solution. Allocating funds from the provincial fund dedicated to the treatment and management of hepatitis and syphilis within the district can have a significant impact. These funds could be utilized to procure essential medications and reagents required for diagnostic and treatment procedures, effectively bridging the gap caused by the current financial limitations. Furthermore, a joint effort between province-level health authorities and the provincial government could help formulate a comprehensive strategy to allocate resources effectively, ensuring that treatment protocols are followed consistently and patient outcomes are improved. This collaborative approach holds the potential to create a sustainable framework for addressing the challenges faced in managing hepatitis and syphilis cases, ultimately contributing to better maternal and child health outcomes across the region.

The involvement of non-governmental organizations (NGOs) in monitoring the treatment of pregnant women with syphilis and hepatitis is required [44]. Currently, the support provided by NGOs has been predominantly directed towards pregnant women with HIV; however, extending this assistance to encompass women with syphilis and hepatitis is required to enhance EMCTC outcomes. Having NGOs provide guidance and support to these women will contribute to better health outcomes and safer pregnancies. NGOs have established effective strategies for ensuring treatment adherence among pregnant women with HIV [45]. By adapting and expanding these strategies to encompass syphilis and hepatitis, a comprehensive and holistic approach to maternal health can be achieved. Furthermore, the inclusion of NGOs in monitoring and supporting pregnant women with syphilis and hepatitis aligns with the broader goal of improving maternal and child health. Ensuring that all pregnant women receive the necessary medical attention and emotional support contributes to the reduction of maternal and neonatal mortality rates.

### 4.3. Study Limitations

This study predominantly examined process indicators and coverages, which provide insight into implementing intervention but do not capture the impact on targets because of the lack of data on the diagnosis of exposed infants, resulting in the unavailability of data related to the program’s effects on infants. Lastly, the study’s mixed methods design may have inherent data integration and interpretation limitations. Despite these limitations, this study provides valuable insight into the progress and challenges of eliminating mother-to-child transmission of HIV, syphilis, and hepatitis B in Bali Province, informing future interventions and strategies. Moreover, this study used secondary data provided by the health office collected by PHC; however, data on the epidemiological variables, such as socio-economic status, demographic, and behavioral factors were limited, which limited our ability to conduct a more in-depth analysis of the associations between these variables and vaccination coverage. Therefore, further study to assess and explore the factors influencing triple EMCTC implementation is recommended.

## 5. Conclusions

There a several points in the study conclusions that can be highlighted. First, the syphilis and hepatitis B screening coverage showed significant improvements, particularly in Buleleng District, indicating progress in the EMTCT; however, there was decreasing adequate treatment coverage for pregnant women with syphilis in Badung and Denpasar. Second, data on hepatitis B treatment were unavailable, highlighting the need for further investigation and monitoring. Adequate treatment coverage for pregnant women with syphilis experienced fluctuations across the districts. ART treatment coverage for women with HIV showed an increasing coverage in certain districts, but challenges persist in achieving the minimum WHO coverage requirement. Third, hepatitis B vaccination coverage exceeded the WHO target for preventing mother-to-child transmission in all three districts, indicating progress in vaccination efforts. Finally, this study identified challenges in eliminating mother-to-child transmission, including suboptimal utilization of information systems, limited involvement of the private sector in screening efforts, and a lack of standardized referral procedures for pregnant women with hepatitis B. These challenges require attention and targeted interventions to improve overall outcomes.

Therefore, treatment for pregnant women with Hepatitis B must be aligned with WHO recommendations, and monitoring and evaluation for a few more years are still necessary to declare the success of eliminating the transmission of HIV, syphilis, and hepatitis B from mother to child. Information systems need to be enhanced to ensure data quality in calculating the elimination of these three infectious diseases from mother to child. A robust information system can assist in collecting, monitoring, and reporting data related to HIV, syphilis, and hepatitis B cases in pregnant women, as well as the actions taken. This enables healthcare professionals and policymakers to monitor and evaluate the program’s success, identify potential issues, and take necessary actions to enhance the effectiveness of the elimination program. Furthermore, there is a need to improve training and education for medical staff and healthcare workers involved to ensure a good understanding of the latest procedures and guidelines in managing and eliminating HIV, syphilis, and hepatitis B from mother to child. With a firm understanding, they can provide appropriate and accurate services to infected pregnant women and contribute to achieving the elimination goals.

## Figures and Tables

**Table 1:** Conceptual definition and process indicators for triple EMTCT.

No.	Process Indicator	Measurement
1.	Coverage of prenatal examinations during the first visit.	The number of pregnant women who undergo their first antenatal examination at healthcare facilities, regardless of gestational age, divided by the total number of pregnant women in the target population for the reporting year, multiplied by 100.
2.	Coverage of HIV testing in pregnant women.	The number of pregnant women tested for HIV divided by the total number of women in the target population who attended ANC visits, multiplied by 100%.
3.	The proportion of pregnant women who test positive for HIV.	The number of pregnant women who test positive for HIV among the total number of pregnant women tested for HIV during ANC visits multiplied by 100%.
4.	The number of pregnant women with HIV who receive/initiate antiretroviral therapy (ARV).	The number of pregnant women living with HIV who receive ARV divided by the number of newly diagnosed pregnant women living with HIV who enter care, multiplied by 100.
5.	Coverage of syphilis testing in pregnant women.	The number of pregnant women tested for syphilis divided by the total number of pregnant women in the target population who attended ANC visits, multiplied by 100%. Screening was conducted using *Treponema pallidum* (TP) rapid and Treponema pallidum hemagglutination (TPHA).
6.	The proportion of pregnant women who test positive for syphilis.	The number of pregnant women diagnosed with syphilis, divided by the total number of pregnant women tested for syphilis, multiplied by 100%.
7.	Coverage of pregnant women with syphilis receiving adequate treatment.	The number of pregnant women with syphilis treated with benzathine penicillin divided by the total number of pregnant women with syphilis, multiplied by 100%.
8.	Coverage of early detection of hepatitis B in pregnant women.	The number of pregnant women tested for hepatitis B divided by the total number of pregnant women in the target population who attended ANC visits, multiplied by 100%.
9.	Coverage of treatment for pregnant women with hepatitis B.	The number of pregnant women with hepatitis B and high viral load treated with tenofovir divided by the total number of pregnant women with hepatitis B, multiplied by 100%.
10.	The proportion of pregnant women who test positive for hepatitis B.	The number of pregnant women who test positive for hepatitis B divided by the total number of pregnant women tested for hepatitis B during ANC visits, multiplied by 100%.
11.	Coverage of newborns from hepatitis B-positive mothers receiving HBV vaccination (HB0) and hepatitis B immunoglobulin (HBIG) within 24 h of birth.	The number of newborns from hepatitis B-positive mothers who received HB0 and HBIg < 24 h after birth divided by the number of babies born to hepatitis B-positive mothers in the same time period, multiplied by 100%
12.	Coverage of infants who receive complete three-dose hepatitis B immunization (HB0–HB3).	The number of infants who receive complete hepatitis B immunization divided by the target number of infants during a specific period, multiplied by 100%.

Note: Antenatal care (ANC); human immunodeficiency virus (HIV).

**Table 2:** Average prevalence of HIV, syphilis, and hepatitis B among pregnant women in the years 2019–2022.

Location/Year	HIV		Syphilis		Hepatitis B	
	Pregnant Women Screening Coverage (%)	The Proportion of Pregnant Women Who Test Positive	Pregnant Women Screening Coverage (%)	The Proportion of Pregnant Women Who Test Positive	Pregnant Women Screening Coverage (%)	The Proportion of Pregnant Women Who Test Positive
Denpasar						
2019 (n = 17,290)	93.12	0.30	82.88	0.32	86.39	1.32
2020 (n = 12,493)	86.74	0.13	77.17	0.79	94.84	1.48
2021(n = 17,306)	69.48	0.33	75.17	0.73	70.39	0.88
2022 (n = 18,161)	68.09	0.22	75.67	0.74	73.77	0.53
Average	79.36	0.24	77.72	0.64	81.36	1.05
Badung						
2019 (n = 12,358)	95.53	0.31	85.90	1.10	88.33	1.39
2020 (n = 11,199)	85.73	0.34	72.25	0.75	84.32	1.03
2021 (n = 10,997)	97.82	0.27	87.24	0.61	88.00	1.09
2022 (n = 10,084)	94	0.16	89	0.36	89.00	0.65
Average	93.27	0.27	85.37	0.70	87.41	1.04
Buleleng						
2019 (n = 11,375)	87.92	0.37	60.44	0.52	29.03	2.88
2020 (n = 12,259)	86.74	0.42	83.31	0.68	82.84	1.19
2021 (n = 10,962)	92.00	0.31	93.99	0.64	94.28	1.07
2022 (n = 10,676)	96.00	0.24	86.98	0.37	95.35	1.03
Average	90.66	0.33	81.18	0.55	75.37	1.54

Note. n = total of pregnant women (ANC).

**Table 3:** Evaluation of indicator processes in Denpasar, Badung, and Buleleng Districts from 2019 to 2022.

Indicators	Denpasar				Badung				Buleleng			
	2019	2020	2021	2022	2019	2020	2021	2022	2019	2020	2021	2022
EMTCT Process Targets												
≥95% ANC coverage (at least one visit(ANC-1))	99.90	72.18	96.89	99.89	115.56	104.72	88.16	84.05	104.10	112.19	102.02	97.07
≥95% coverage of HIV testing for pregnant women	93.12	76.58	77.17	94.84	96.53	85.73	84.93	90.59	87.92	86.74	92.35	96.46
≥95% coverage of syphilis testing of pregnant women in ANC	82.88	69.48	75.67	70.39	85.90	72.25	87.24	89.47	60.44	83.31	93.99	86.98
≥90% coverage of HBsAg antenatal testing among pregnant women	86.39	68.09	77.07	73.73	88.33	84.32	87.66	89.47	29.03	82.84	94.28	95.35
Maternal Treatment												
≥95% ART coverage of pregnant women living with HIV	100	100	100	81.08	100	100	100	100	72.97	97.78	83.87	100
Adequate syphilis treatment of syphilis-seropositive-pregnant women of ≥95%	100	68.12	95.79	71.28	62.39	93.36	100	69.69	83.33	71.01	78.79	100
≥90% coverage with antivirals for eligible HBsAgG-positive pregnant women with high viral loads	n.a	n.a	n.a	n.a	n.a	n.a	n.a	n.a	n.a	n.a	n.a	n.a
Infant HBV Vaccination												
≥90% coverage with three doses of HBV infant vaccinations (HepB3)	97.78	95.5	98.2	98.3	105.5	85.64	94.1	103.25	105	114.01	92.63	96.07
≥90% coverage of HBV-exposed babies with hepatitis B immune globulin (HBIg)	96	97.18	100	100	100	100	100	100	95	100	100	100
≥90% HepB timely birth dose coverage (with the universal program) or infants at-risk (with targeted timely HepB-BD)	100	100	100	100	100	100	100	100	101.5	121.45	119.63	103.34

Note: n.a. (no available data).

**Table 4:** Themes and quotations.

Aspect	Theme	Quotations
Data	Monitoring and evaluation	Q1: “We conduct monitoring and evaluation every three months at the district and provincial levels, as well as validate data against reports from each healthcare facility, including both primary health centers and hospitals.” (R1, in-depth interview) Q2: “Every month, specialist obstetricians and gynecologists visit the community health centers (Puskesmas), and the program holders conduct internal monitoring and evaluation at each Puskesmas.” (R12, FGD)
	Information system	Q3: “...we haven’t been consistent with using the e-cohort because not everyone is actively using it, and the e-cohort was only introduced in 2022, so we still rely on manual reporting using Excel spreadsheets and recording in other books, using different systems, which leads to duplication of work.” (R2, FGD) Q4: “The existing information system, SIHA, has been in place for a long time, but it is only used for recording purposes and is not capable of case tracking.” (R6, in-depth interview)
	Data resources	Q5: “The data we receive is comprehensive. Midwives, specialist doctors, and general practitioners who provide maternal and child healthcare are required to report to us and input data into the e-cohort. This applies to both public and private sector.” (R3, in-depth interview) Q6: “All midwives and doctors in our primary healthcare area are required to submit service data to us every month, as well as private hospitals.” (R22, FGD)
	Resources	Q7: “...for laboratory examinations, I conduct them as a laboratory analyst using RDT (Rapid Diagnostic Test), and the results are available in less than 30 min.” (R10, FGD) Q8: “We are required to have laboratory analysis, and all our counselors have received counseling training.” (R2, in-depth interview)
Laboratory	The availability of the laboratory	Q9: “The HBV DNA testing has not been conducted as not all healthcare facilities have the equipment, and even if we can, the cost is very high and not covered by the national health insurance (BPJS).” (R4, in-depth interview) Q10: “In our primary health centers, we only conduct serological tests. For CD4 testing and titer checks, patients need to go to general hospitals...” (R23, FGD)
	The availability of reagents and drugs	Q11: “Our coverage is not the same for HIV, Syphilis, and Hepatitis B screening because we often experience a shortage of Syphilis and Hepatitis B reagents, as is the case this month, where the Syphilis reagent is unavailable. The availability of Benzathine Penicillin is also often insufficient from the central supply.” (R1, FGD) Q12: “The procurement of reagents is done centrally using funds from the national budget (APBN), and at the provincial level, there has been a shortage of reagents for syphilis and hepatitis B for the past three months...” (R25, FGD)
Program	Policy	Q13: “Pregnant women with asymptomatic Hepatitis B, such as jaundice, are referred to obstetricians/gynecologists. However, if they exhibit symptoms of jaundice, we refer them to specialist doctors in internal medicine...” (R6, in-depth interview) Q14: “We always adhere to the policies set by the Ministry of Health, but the district health office regulates the treatment flow and referrals for infected individuals...” (R15, FGD)
	Involvement of the non-public sector	Q15: “...for the screening of these three diseases, we conduct them at the primary health centers, and if a person tests positive, we refer them to the hospital.” (R5, FGD) Q16: “All screening procedures are conducted at the primary health centers (puskesmas), so midwives and village midwives must refer their patients to the nearest puskesmas.” (R17, FGD)
Human rights	Human rights	Q17: “...for the initial information, we provide it verbally and obtain informed consent. If someone tests positive, we conduct counseling and make a referral. If they disagree, a refusal form needs to be filled out.” (R12, FGD) Q18: “We provide counseling to all pregnant women who test positive before their referral or treatment. Almost all of them accept the treatment, especially when they are pregnant...” (R19, FGD)

## Data Availability

Not applicable.
